# Correction: Sulphonated graphene oxide catalyzed continuous flow synthesis of pyrazolo pyrimidinones, sildenafil and other PDE-5 inhibitors

**DOI:** 10.1039/d2ra90133a

**Published:** 2023-01-06

**Authors:** Vinay Kumar Sthalam, Bhushan Mahajan, Purushotham Reddy Karra, Ajay K. Singh, Srihari Pabbaraja

**Affiliations:** a Department of Organic Synthesis and Process Chemistry, CSIR-Indian Institute of Chemical Technology Hyderabad 500007 India srihari@iict.res.in; b Academy of Scientific and Innovative Research (AcSIR) Ghaziabad-201002 India

## Abstract

Correction for ‘Sulphonated graphene oxide catalyzed continuous flow synthesis of pyrazolo pyrimidinones, sildenafil and other PDE-5 inhibitors’ by Vinay Kumar Sthalam *et al.*, *RSC Adv.*, 2022, **12**, 326–330, https://doi.org/10.1039/D1RA08220E.

The authors regret that the one of the affiliations (affiliation *b*) was incorrectly shown in the original manuscript. The corrected list of affiliations is as shown here.

The Royal Society of Chemistry apologises for these errors and any consequent inconvenience to authors and readers.

## Supplementary Material

